# An Antenna Proximity Sensor for Mobile Terminals Using Reflection Coefficient

**DOI:** 10.3390/s18072103

**Published:** 2018-06-30

**Authors:** Wonsub Lim, Dongil Yang, Youngoo Yang

**Affiliations:** 1 College of Information and Communication Engineering, Sungkyunkwan University, 2066 Seobu-ro, Jangan-gu, Suwon, Gyeonggi-do 16419, Korea; wonsub.lim@samsung.com; 2 Mobile Communication Division, Samsung Electronics Ltd., 129 Samsung-ro, Yeongtong-gu, Suwon, Gyeonggi-do 16677, Korea; dongil.yang@samsung.com

**Keywords:** antenna sensor, proximity sensor, reflection coefficient, mobile terminals, smartphone, LTE signal

## Abstract

This paper presents a new antenna proximity sensor for mobile terminals based on the measured reflection coefficient using a bidirectional coupler which is positioned between the main antenna and the front-end module. Using the coupled forward and reverse long-term evolution signals by the bidirectional coupler, the reflection coefficient looking into the antenna was calculated in the base-band processor. The measured reflection coefficients showed clear differences for both the types of objects, and the distances between the terminal and the objects. The proposed antenna proximity sensor showed a recognition distance that was approximately 5 mm longer than that of a conventional capacitive proximity sensor.

## 1. Introduction

As the technologies for mobile terminals evolve, many types of sensors have been deployed in the terminals [1,2,3,4,5,6,7,8,9,10,11,12,13,14,15,16,17]. These sensors, which include acceleration sensors [4], gyroscope sensors [5], magnetic sensors [6], light sensors, touch sensors [7], fingerprint sensors [8], temperature sensors [9,10], barometer sensors [11], heart rate sensors [12,13], iris scanners [14], and proximity sensors, have all been widely used because they provide a high level of user convenience [15,16,17]. Among the many sensors for mobile terminals, the proximity sensor, which senses the distance between the terminal and external objects, is one of the most essential sensors for various applications [17].

A proximity sensor can be classified as either an ultrasonic proximity sensor, an inductive proximity sensor, or a capacitive proximity sensor according to the detection principle used. An ultrasonic sensor contains an ultrasonic transceiver, which transmits an ultrasonic signal and receives the reflected signal back from objects within a limited distance. The distance between the sensor and the object can be obtained by multiplying the velocity of the ultrasonic wave by the time the wave takes to make a round-trip [18].

The inductive proximity sensor is mainly used to detect metals. An oscillator in the sensor generates a sinusoidal current that has a fixed magnitude. As the distance between the metal surface and the sensor decreases, the eddy current becomes larger and, as a result, the amplitude of the oscillating current decreases. If the amplitude of the oscillating current becomes lower than the threshold, the trigger circuit will determine that a metal object is present [19].

The capacitive proximity sensor senses not only metals but also non-metals by measuring capacitance. As an object approaches the proximity sensor, the sensor can detect the object using the measured capacitance from the sensor. Currently, capacitive proximity sensors are widely used to recognize the hand-gripping condition of the mobile terminals. This is done by connecting a sensing probe from the sensor integrated circuit to the main antenna, which could deteriorate the radiation performance [20]. The capacitive proximity sensor also takes space on the printed circuit board (PCB) [21]. In addition, since capacitance sensors return only the voltage as a result of measuring the capacitance, object types cannot be recognized [22].

In this paper, a new proximity sensor is proposed based on the measured reflection coefficient using a bidirectional coupler between the front-end module (FEM) and the antenna. Some object types can also be recognized using the phase information of the reflection coefficient. The overall performances will be presented to validate its sensing ability as a proximity sensor.

## 2. Principle of the Antenna Proximity Sensor

**Figure 1:**
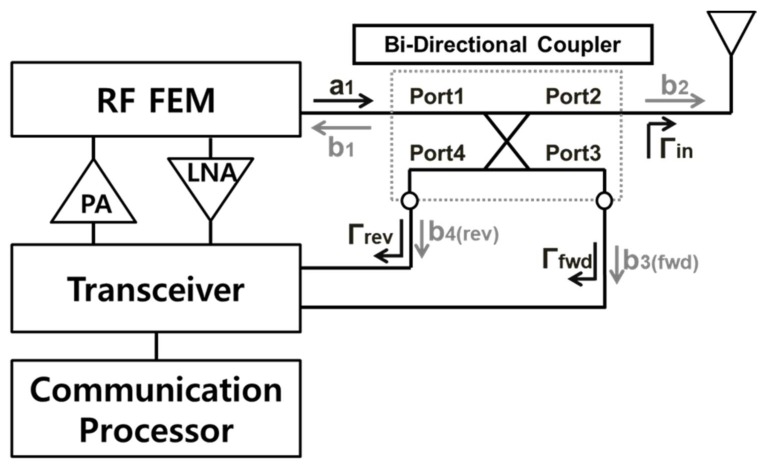
Block diagram of the antenna proximity sensor.

Figure 1 shows a block diagram of the antenna proximity sensor. The baseband signal is generated by the communication processor (CP) and is applied to the transceiver. Then, the transceiver up-converts the signal into the RF band which is radiated by the antenna through the power amplifier (PA) and RF FEM. A bi-directional coupler is located between the RF FEM and the antenna.

The 4-port S-parameter of the bidirectional coupler is used to find the reflection coefficient looking into the antenna. The 4-port S-parameter of the bidirectional coupler is given by:

(1) $$\left[\begin{array}{c} b_{1}\\ b_{2}\\ b_{3}\\ b_{4} \end{array}\right]=\left[\begin{array}{cccc} S_{11} & S_{12} & S_{13} & S_{14}\\ S_{21} & S_{22} & S_{23} & S_{24}\\ S_{31} & S_{32} & S_{33} & S_{34}\\ S_{41} & S_{42} & S_{43} & S_{44} \end{array}\right]\left[\begin{array}{c} a_{1}\\ b_{2}\cdot \Gamma_{in}\\ b_{3}\cdot \Gamma_{fwd}\\ b_{4}\cdot \Gamma_{rev} \end{array}\right]$$

where $a_{n}$ and $b_{n}$ are the incident and reflected signals for the *n*-th port of the coupler, respectively. $\Gamma_{in}$ is the input reflection coefficient of the antenna. $\Gamma_{fwd}$ and $\Gamma_{rev}$ are the input reflection coefficients for the forward and reverse ports of the coupler, respectively. $b_{3}$ and $b_{4}$ can be obtained from (1) as follows:

(2) $$b_{3}=S_{31}a_{1}+S_{32}b_{2}\Gamma_{in}+S_{33}b_{3}\Gamma_{fwd}+S_{34}b_{4}\Gamma_{rev}$$

(3) $$b_{4}=S_{41}a_{1}+S_{42}b_{2}\Gamma_{in}+S_{43}b_{3}\Gamma_{fwd}+S_{44}b_{4}\Gamma_{rev}$$

Since the forward and reverse ports of the bidirectional coupler are in good match to the transceiver through 50 Ω, $b_{3}\Gamma_{fwd}$ and $b_{4}\Gamma_{rev}$ in (2) and (3) can be approximated as zero. It is assumed that if the isolation between the ports and the directivity of the coupler are large enough, $b_{3}$ and $b_{4}$ can be approximated as follows:

(4) $$b_{3}\approx S_{31}a_{1}$$

(5) $$b_{4}\approx S_{42}S_{21}a_{1}\Gamma_{in}$$

By replacing $a_{1}$ in (5) with $b_{3}/S_{31}$ from (4), the input reflection coefficient of the antenna can be derived as follows:

(6) $$\frac{b_{4}}{b_{3}}\approx \frac{S_{42}S_{21}}{S_{31}}\Gamma_{in}$$

(7) $$\Gamma_{in}\approx \frac{S_{31}b_{4}}{S_{42}S_{21}b_{3}}=\frac{b_{4}}{S_{21}b_{3}}$$

where $S_{31}$ and $S_{42}$ are the same. Since we know $S_{21}$ of the coupler, the approximated value of $\Gamma_{in}$ can be obtained from the ratio of the reverse coupled signal to the forward coupled signal [23].

## 3. Sensing Procedure

**Figure 2:**
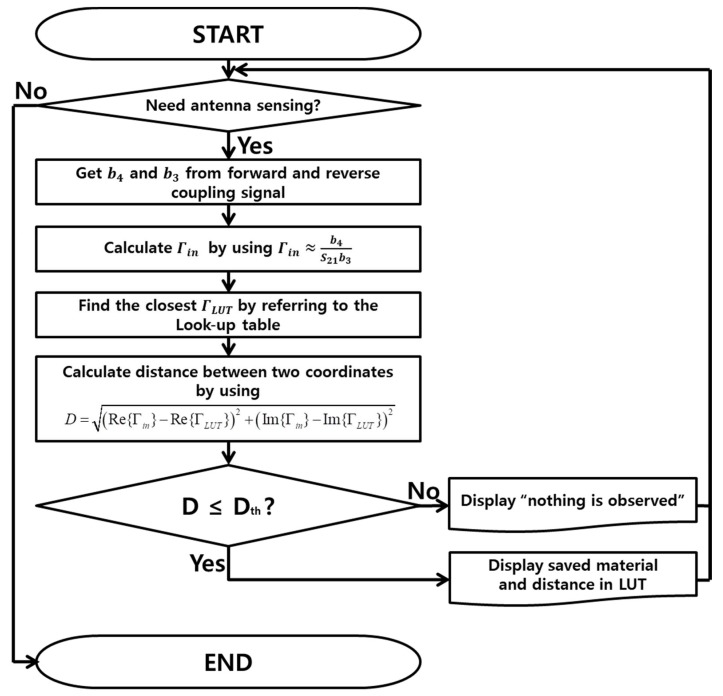
Procedure to determine the object type and the distance.

Figure 2 shows the computational procedure used to determine the object type and the distance using the extracted input reflection coefficient of the antenna. First, the mobile terminal determines whether it is necessary to use the antenna proximity sensor for reasons such as antenna impedance tuning or power control under the hand-gripping condition. If it is necessary, the mobile terminal senses $b_{3}$ and $b_{4}$ from the forward and reverse ports of the coupler and calculates the reflection coefficient by using (7). The distances between the measured reflection coefficient and the reflection coefficients stored in the look-up table (LUT) are calculated using the following equation.

(8) (Re{Γin}−Re{ΓLUT})2+(Im{Γin}−Im{ΓLUT})2

where $\Gamma_{LUT}$ is the pre-stored reflection coefficients for the various distances and material types in the LUT. From the calculated distances, the nearest point in the LUT can be found. If the distance for the nearest point is no longer than the distance threshold ($D_{th}$), the distance and material type assigned to the point in the LUT will be returned. Otherwise, “nothing is detected” will be returned.

## 4. Measurement Results

**Figure 3:**
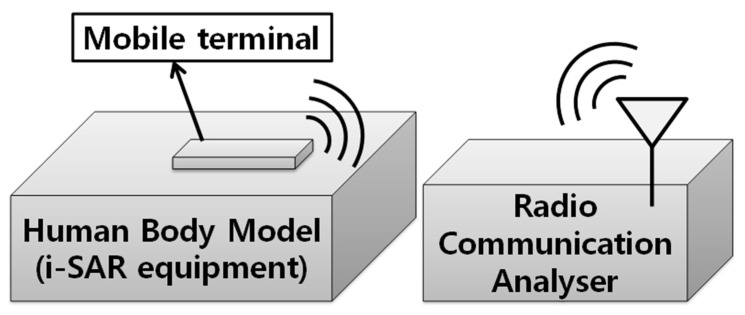
Test setup for the antenna proximity sensor.

Figure 3 shows the test setup for the antenna proximity sensor. The setup consists of a radio-communication analyzer, Anritsu’s MT8820C, and a phantom model (Speag’s cSAR3D) which is the equivalent of using human tissues for testing purposes. The 847 MHz band long-term evolution (LTE) signal, based on a quadrature phase-shift keying (QPSK) with a signal bandwidth of 20 MHz and 10 resource blocks, was applied for the test. The distance between the phantom model and the mobile terminal was changed from 0 mm to 10 mm, in 1 mm increments.

**Figure 4:**
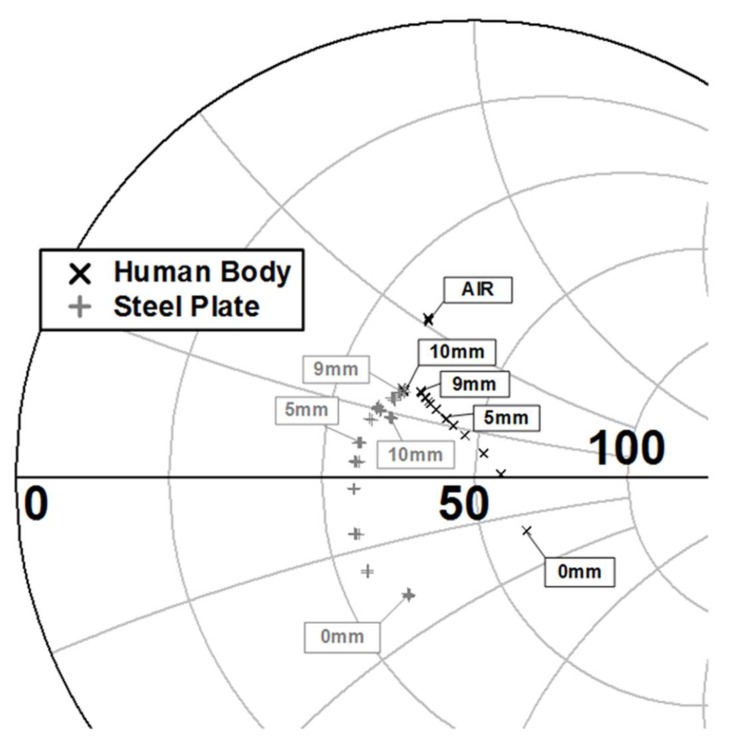
Measured $\Gamma_{in}$ for the human body and the steel plate according to the distances.

Figure 4 shows the measured $\Gamma_{in}$ according to the distance between the mobile terminal and the object. As the distance gets smaller, the difference in the measured reflection coefficients between the human body and the steel plate gets larger. The measured reflection coefficients, which are still far from the reflection coefficient with no object nearby, become almost the same at a distance of 10 mm. The measured results show that the object type and the distance can be simultaneously identified for the distance range of from 0 to about 8 mm.

**Figure 5:**
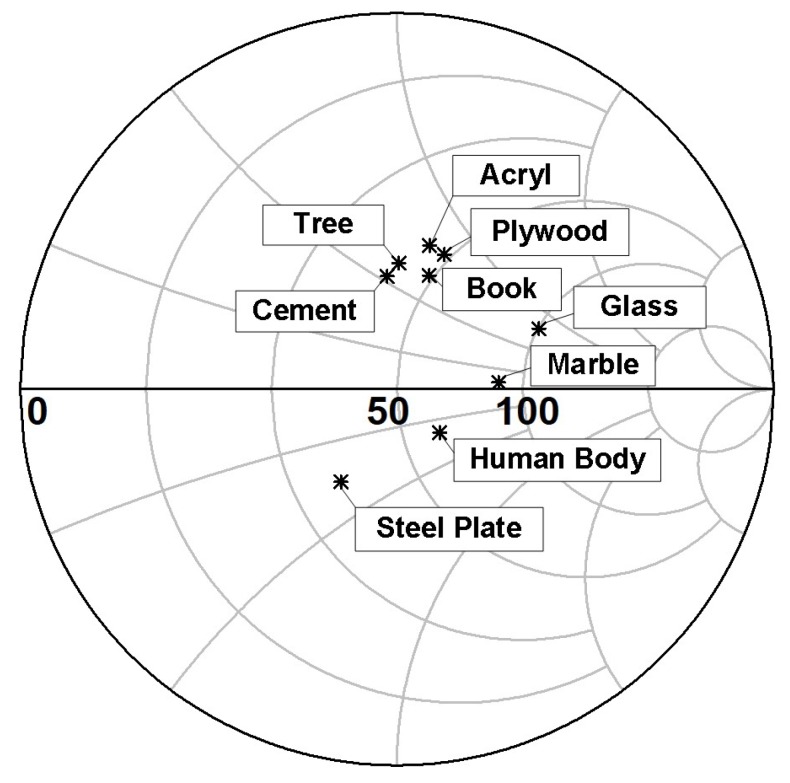
Measured $\Gamma_{in}$ according to the object types.

Figure 5 shows the measured according to the object types at a distance of 0 mm. Very different reflection coefficients can be measured, as shown with the steel plate, human body, marble, and glass. This result shows that the antenna proximity sensor can also be used as a sensor to find some object types.

**Figure 6:**
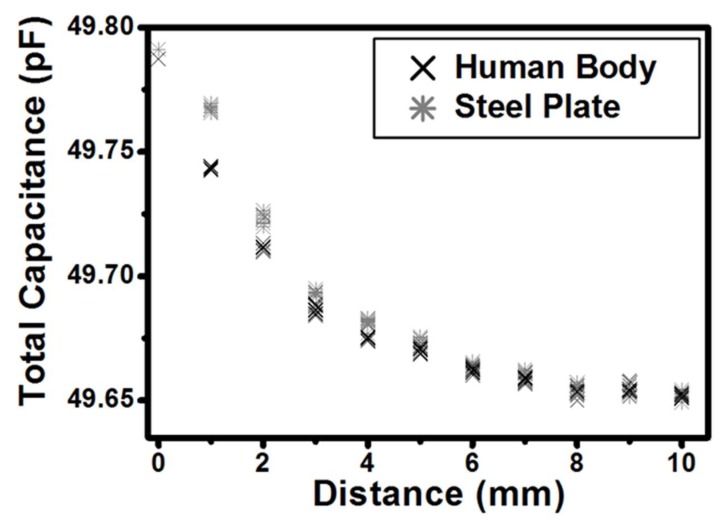
Characteristics of the conventional capacitance sensor.

Figure 6 shows the measured capacitances from the conventional capacitance sensor, Semtech’s SX9310, according to the distance between the mobile terminal and the object. As shown, since the conventional capacitance sensor returns very similar values for both the human body and the steel plate, it cannot be used to distinguish between object types. It also becomes more difficult to estimate the exact distance as the distance increases beyond 5 mm. Table 1 shows the summary of the measurement results for the conventional capacitance sensor and the proposed antenna proximity sensor.

## 5. Conclusions

An antenna proximity sensor, which is based on the measured reflection coefficient using a bidirectional coupler between the FEM and the antenna, is proposed for use on mobile terminals. The operational principles and the sensing procedures have been explained. The proposed proximity sensor was implemented and was experimentally verified using an LTE signal.

The experimental results show that the proposed sensor can recognize the objects in the distance range of 0 to about 8 mm using the object identification function and can recognize the objects at a distance of up to about 10 mm using the distance identification function. The proposed sensor was experimentally proven to be superior to the conventional capacitance sensor in performance, function, cost, and size.

## Figures and Tables

**Table 1:** Summary of the measurements.

Sensor Type	Sensing Distance	Condition
Conventional capacitance sensor (Semtech’s SX9310)	5 mm	No object identification
Proposed antenna proximity sensor	8 mm	Object identification
	10 mm	No object identification
